# Characterization of the Synergistic Antioxidant Activity of Epigallocatechin Gallate (EGCG) and Kaempferol

**DOI:** 10.3390/molecules28135265

**Published:** 2023-07-07

**Authors:** Qiang Zhang, Junkun Pan, Hui Liu, Zhonggao Jiao

**Affiliations:** Zhengzhou Fruit Research Institute, Chinese Academy of Agricultural Sciences, Zhengzhou 450009, China; zhangqiang02@caas.cn (Q.Z.); zqlyf0@163.com (J.P.)

**Keywords:** EGCG, kaempferol, synergistic effect, cellular antioxidant activity, antioxidant enzymes

## Abstract

Epigallocatechin gallate (EGCG) and kaempferol exhibit cellular antioxidant activity; however, their interactive effects in terms of antioxidant actions and underlying mechanisms remain unclear. In this study, their cytoprotective effects were examined against 2,2-azobis (2-amidinopropane) dihydrochloride solution (ABAP)-induced oxidative stress in HepG2 cells. The results showed that the median effective dose (EC_50_) of the EGCG and kaempferol (6:1.5, c/c) combination was 3.4 ± 0.1 μg/mL, with a combination index (CI_avg_) value of 0.54, which represented a significant synergistic effect. Further experiments proved that the combined pretreatment with EGCG and kaempferol exerted protective effects by suppressing reactive oxygen species (ROS) generation, upregulating cellular antioxidant enzyme activities (superoxide dismutase (SOD), catalase (CAT), and glutathione peroxidase (GSH-Px)) in a dose-dependent manner. The mechanism of synergistic antioxidant effects of EGCG combined with kaempferol may be due to the up-regulation of higher antioxidant enzyme activities that improve the antioxidant capacities and balance the cell oxidative stress. The synergistic antioxidant effect of EGCG and kaempferol can provide a theoretical basis for the development of formulas of functional food ingredients.

## 1. Introduction

Reactive oxygen species (ROS) act as signal molecules in the metabolic oxidation/antioxidant balance of the body. However, when an imbalance occurs between the generation and scavenging of ROS, the excessive production of ROS causes oxidative stress, leading to damage to biomolecules such as DNA, lipids, and proteins. ROS play an important role in the diseases in humans, such as cancer, coronary heart diseases, and aging [1,2,3]. To avoid oxidative damage, the body can produce endogenous antioxidants and enzymes, such as phenolics, flavonoids, and carotenoids, to catalyze the metabolism of ROS and eliminate ROS by exogenous antioxidants. Recently, many studies have reported extensive biological activities of flavonoids and their effects on the prevention and treatment of various diseases [4,5,6]. Dietary flavonoids can increase the antioxidant capacity and scavenge ROS, leading to restoration of redox homeostasis [7,8,9]. Numerous studies have begun to focus on the food system with various co-existing flavonoids owing to health benefits of the combined effects [10,11,12]. Therefore, various studies with different models have been applied to explore the mechanisms of flavonoids combinations [13,14,15,16]. This study is based on this phenomenon, focusing on the synergistic effects of key flavonoids.

**Figure 1:**
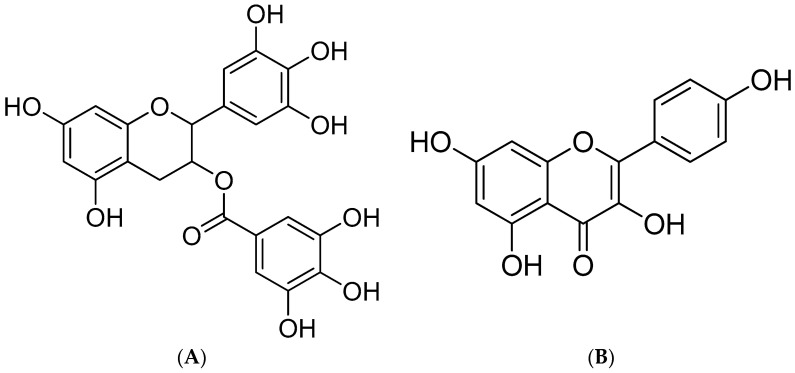
Structure of EGCG (**A**) and kaempferol (**B**).

Flavonoids are some of the most abundant phenolic compounds in various fruits, vegetables, grains, spices, beverages, and medicinal plants, which are structured by a C_6_–C_3_–C_6_ skeleton labeled with the rings A, B, and C. The subclass includes flavones, flavonols, flavanones, flavanols, anthocyanidins, and isoflavonoids. In addition, epigallocatechin gallate (EGCG, MW = 458.38 g/mol) and kaempferol (MW = 286.23 g/mol) (Figure 1) are abundant flavonoids and excellent antioxidants in numerous plant-derived foods, such as apples, blueberries, tea, citrus, and peppers, which possess beneficial effects on human health, including health maintenance and disease prevention [17,18]. They possess multiple hydroxyl groups on the C_6_–C_3_–C_6_ skeleton, which give them strong antioxidant activity. They are an important part of the human diet and have a variety of biological activities such as antioxidant, anti-inflammatory, and antiproliferative effects [19].

Due to the diversity of the human diet, various flavonoids commonly coexist in food or are ingested at the same time in a meal. How these flavonoids affect human health is not yet clear. Studying the interaction between flavonoid components can reveal their physiological functions in the body. Compound association may lead to synergic protective effects at concentrations substantially lower than those used when given separately. Therefore, to evaluate the synergistic compound combinations, the combination index (CI) is utilized. The CI quantitatively depicts synergism (CI < 1), additive effect (CI = 1), and antagonism (CI > 1) [20]. Li et al. found that quercetin with catechin showed synergistic anti-inflammatory effects, which may be due to the inhibition of the activation of TLR-MyD88-mediated NF-kappa B and mitogen-activated protein kinases signaling pathways [20]. The results of a growing number of studies suggest that the combinations of different flavonoids can enhance biological activity and cause synergistic effects. Colon et al. found that the SE (synergistic effect) value of quercetin and EGCG was 1.14 ± 0.03, and the ESC (experimental scavenging capacity) value was significantly higher than the TSC (theoretical scavenging capacity) value in the DPPH assay. Therefore, the antioxidant interaction associated with quercetin and the EGCG mixture was a synergistic interaction in the DPPH assay [21]. Therefore, this study provided a hypothesis that the EGCG and kaempferol combination may show synergistic effects related to the protein expression pathway. Those effects may be possible mechanisms that cause antioxidant interactions on cells.

Therefore, this study aimed to (1) investigate the cellular antioxidant activity (CAA) of the combined EGCG and kaempferol against 2,2-azobis (2-amidinopropane) dihydrochloride solution (ABAP)-induced oxidative stress in HepG2 cells; (2) study the synergistic, antagonistic, and additive interactions resulting from the different ratios of EGCG and kaempferol combination by the Chou–Talalay combination index (CI) method; and (3) elucidate the mechanism underlying the synergistic effect of the EGCG and kaempferol combination on ABAP-mediated oxidative stress by antioxidant enzyme activities (superoxide dismutase (SOD), catalase (CAT), and glutathione peroxidase (GSH-Px)). To summarize, the main target of this work was to know the possible synergistic antioxidant mechanism of EGCG and kaempferol combinations. The aim of this study was to provide theoretical guidance for the use of flavonoids in functional foods ingredient formula development and improve its utilization.

## 2. Results and Discussion

### 2.1. Effects of EGCG and Kaempferol on Cytotoxicity Activity

**Figure 2:**
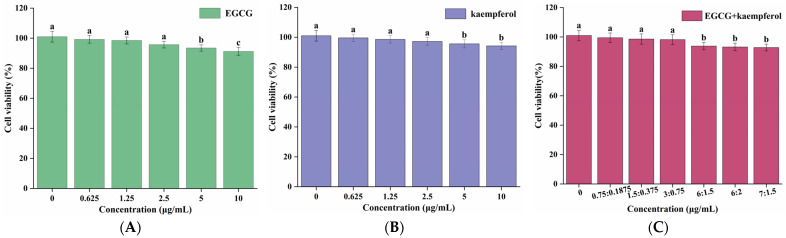
Cell viabilities of HepG2 cells treated with various concentrations of EGCG (**A**), kaempferol (**B**), and EGCG + kaempferol combination (**C**). Data are shown as the mean ± SD from four independent experiments. Different letters represent significant differences at *p* < 0.05.

To explore the cytotoxicity of pretreatment of EGCG, kaempferol, and EGCG and kaempferol combinations on HepG2 cells, the viability rate of HepG2 cells treated with EGCG, kaempferol, and their combinations was evaluated. The CCK-8 method was used to evaluate the cytotoxicity of EGCG, kaempferol, and their combinations in different concentrations [22]. The viability rate of HepG2 cells is presented in Figure 2. As shown in Figure 2, the cell viability rates of EGCG, kaempferol, and their combinations were found to be over 90%, with no cytotoxicity. For EGCG or kaempferol, when the sample concentration was within 5.0 μg/mL, the cell viability did not change with the increase of the sample concentration. However, when the EGCG concentration reached 5.0–10.0 μg/mL, the cell viability significantly decreased (*p* < 0.05), indicating that HepG2 cells had been damaged under this concentration, but the survival rate was still more than 90%, which was regarded as the normal rate for cells under this condition (Figure 2A). The cells, treated with the EGCG and kaempferol combination with different ratios (3:0.75, c/c) or less still survived normally (*p* > 0.05). However, when the concentration of these combinations exceeded the ratio (3:0.75), the cell viabilities were significantly decreased (*p* < 0.05). Moreover, the cell viability did not change with the different ratios (6:1.5, 6:2, 7:1.5, c/c). To summarize, Figure 2A–C show that the cell viabilities of EGCG, kaempferol, and EGCG and kaempferol combinations (6:1.5, 6:2, and 7:1.5) were over 90%. Hence, the results illustrated that the concentrations of EGCG, kaempferol, and EGCG and kaempferol combinations had no cytotoxicity effects on HepG2 cells. This finding suggested that EGCG, kaempferol, and EGCG and kaempferol combinations had no toxic side effects on HepG2 cells within certain concentration ranges.

### 2.2. EGCG and Kaempferol Combination on Cellular Antioxidant Activity

The cellular oxidative stress due to ROS generated by ABAP was measured spectrofluorometrically using the 2′,7′-dichlorfluorescin diacetate (DCFH–DA) method. DCFH-DA diffuses through the cell membrane and is enzymatically hydrolyzed by intracellular esterase to non-fluorescent DCF, which can be rapidly oxidized to highly fluorescent DCF in the presence of ROS. Hence, the level of cellular fluorescence indicates cellular antioxidant capacity [23].

**Figure 3:**
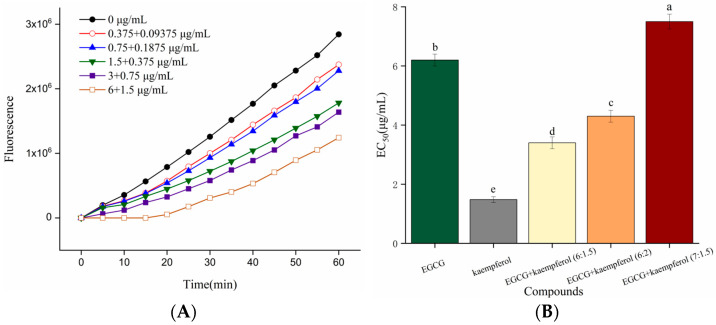
Kinetic curves of ABAP-induced DCF fluorescence and the inhibition of oxidation by the EGCG and kaempferol (6:1.5) combination (**A**) on the fluorescence in HepG2 cells with PBS wash. The EC_50_ of EGCG, kaempferol, and EGCG + kaempferol combinations (**B**). Different letters represent significant differences at *p* < 0.05.

As shown in Figure 3A, the DCF fluorescence values of HepG2 cells, generated from the oxidation caused by ABAP-induced peroxyl radicals, were decreased in the presence of the EGCG and kaempferol combination. The increase in fluorescence from DCF formation was inhibited by the EGCG and kaempferol combination (6:1.5) in a dose-dependent manner, as demonstrated by the curves generated from cells treated with the combination. The cellular antioxidant activities of EGCG, kaempferol, and their combinations were assessed at non-cytotoxic concentrations with phosphate buffer solution (PBS) wash protocols. Figure 3B shows that the median effective dose (EC_50_) of EGCG and kaempferol were 6.2 ± 0.2 and 1.48 ± 0.1 μg/mL, respectively, which were significant (*p* < 0.05). In this study, a combination assay using the Chou–Talalay method was carried out to investigate whether the EGCG and kaempferol combination could work synergistically. For specific combination assays, the EC_50_ values of EGCG and kaempferol were used as a guide to choose individual concentrations. Each combination was prepared at a ratio of (EC_50_)_1_/(EC_50_)_2_ so that each compound contributed equally to the antioxidant effect. Moreover, the other concentration ratios of combinations were slightly modified according to the (EC_50_)_1_/(EC_50_)_2_ to obtain a range of CI values. Hence, the concentration ratios of the combination were 6:1.5, 6:2, and 7:1.5 (c/c), respectively. As shown in Figure 3B, the EGCG and kaempferol combinations (6:1.5, 6:2, c/c) displayed good cellular antioxidant activity and had significant differences (*p* < 0.05) compared with EGCG or kaempferol alone. The EC_50_ of EGCG and kaempferol combinations were 3.4 ± 0.1 (6:1.5), 4.3 ± 0.2 (6:2), and 7.5 ± 0.2 (7:1.5) μg/mL, respectively. The EGCG and kaempferol combination (6:1.5) showed better cellular antioxidant activity (*p* < 0.05) than other combinations. The EC_50_ values of combinations were 0.55-, 0.69-, 1.2-fold of that in the EGCG group, respectively. This showed that the ROS scavenging ability of combinations (6:1.5, 6:2) was significantly higher than that of EGCG. However, the EC_50_ values of combinations were significantly higher (*p* < 0.05) than that in the kaempferol group, indicating that the ROS scavenging ability was weaker than that of kaempferol.

Based on the above results, the EGCG and kaempferol combinations (6:1.5, 6:2, c/c) inhibited ROS generation to protect HepG2 cells from ABAP-induced oxidative damage. Among them, the EGCG and kaempferol combination (6:1.5) showed stronger antioxidant activity. Similar results could be observed in the study of flavonoid combinations. Saw et al. discovered that low concentrations of quercetin + kaempferol, kaempferol + pterostibene, and pterostilbene + quercetin combinations exhibited synergistic effects in protecting HepG2-C8 cells against oxidative stress by increasing NAD(P)H dehydrogenase [quinone] 1 (NQO1) and superoxide dismutase 1 (SOD1) protein expression [24]. Wang et al. found that a quercetin and catechin combination up-regulated the expression of let-7a-5p and miR-25–3p to inhibit the BACH1 activity, which could promote ROS accumulation and inhibit cell growth in HepG2 cells [7]. Thus, our results of the cellular antioxidant activity assay suggest that combinations of EGCG and kaempferol may have potential uses as antioxidants as well, which needs further investigation.

### 2.3. Synergistic Effect of EGCG and Kaempferol Combination

The combination index (CI) method was introduced by Chou and Talalay to quantitatively describe drug combination effects as synergism (CI < 1), additive effect (CI = 1), or antagonism (CI > 1). With the development of Chou–Talalay theory, the CompuSyn software for dose–effect analysis, CI calculation, and Fa–CI plot simulation was developed [24]. In this study, a combination assay using the Chou–Talalay method was carried out to investigate whether the EGCG and kaempferol combinations could work synergistically. For specific combination assays, the EC_50_ values of EGCG and kaempferol were used as a guide to choose individual concentrations. The CI value was evaluated by CompuSyn software using the following formula: CI = (D)_1_/(D_X_)_1_ + (D)_2_/(D_X_)_2_, in which (D_X_)_1_ and (D_X_)_2_ are the concentrations of compound **1** and compound **2**, respectively, where the cellular antioxidant effect is x%. Similarly, (D)_1_ and (D)_2_ show the respective dose of compound **1** and compound **2** in which their combination antioxidant activity is x%. The CI values of the EGCG and kaempferol combination at 50%, 75%, and 90% antioxidant effect (GI_50_, GI_75_, and GI_90_) are shown in Table 1.

**Figure 4:**
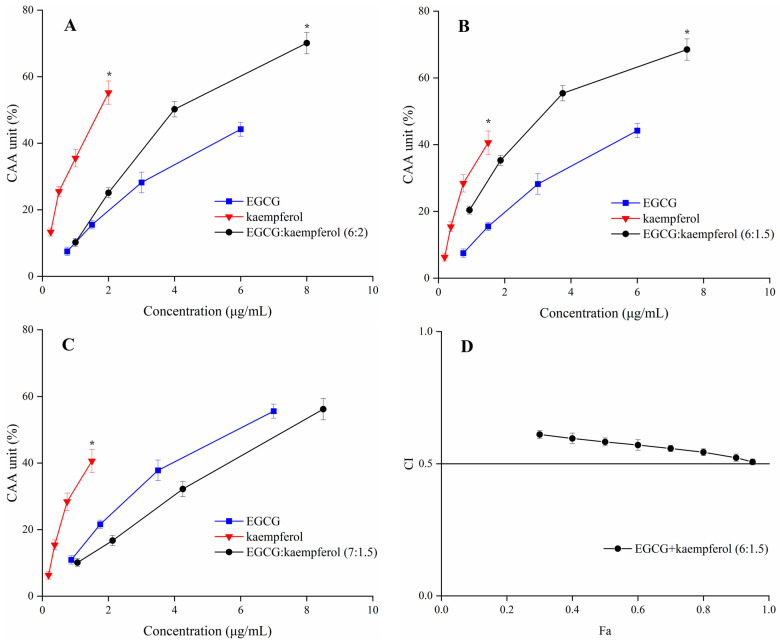
The CAA values of EGCG, kaempferol, and EGCG + kaempferol combinations at different proportions (**A**–**C**); (**D**) the Fa–CI plot of EGCG + kaempferol (6:1.5). * *p* < 0.05.

The CAA units of EGCG, kaempferol, and their combinations are displayed in Figure 4. The tested ratios of EGCG and kaempferol combinations were 6:2, 6:1.5, and 7:1.5. The cellular antioxidant activity of EGCG, kaempferol, and their combination showed a dose–effect relationship. For the combinations (Figure 4A–C), when the sample concentration was within 1.0–2.0 μg/mL, the CAA unit of kaempferol was significantly higher (*p* < 0.05) than EGCG and EGCG and kaempferol combinations. Moreover, when the sample concentration reached 2.0–8.0 μg/mL, the CAA unit of the EGCG and kaempferol combination (6:1.5) was higher (*p* < 0.05) than that of EGCG or kaempferol. Similarly, when the sample concentration reached 8.0 μg/mL, the CAA unit of the EGCG and kaempferol combination (6:2) was 70.1 ± 3.2%, showing a significant increase (*p* < 0.05) with respect to kaempferol. However, when the sample concentration within 2.0–8.5 μg/mL, the CAA unit of the EGCG and kaempferol combination (7:1.5) was lower (*p* > 0.05) than that of kaempferol. The results indicated that the EGCG and kaempferol combinations (6:2, 6:1.5) may possess synergistic effects.

According to the CAA unit of each compound and their combinations at the corresponding concentration, the CI values of the EGCG and kaempferol combinations were calculated by CompuSyn software [25]. The EGCG and kaempferol combination (6:1.5, 6:2) displayed synergistic effects, having the weighted average CI (CI_avg_) values all below 0.9. The combination (6:1.5) exhibited the strongest synergy at different levels of antioxidant activity (GI_50_, GI_75_, and GI_90_; Table 1), which resulted in a CI_avg_ of 0.54. The combination (6:2) was also able to work synergistically (CI_avg_ = 0.85). The CI_avg_ of the EGCG and kaempferol combination (6:1.5) was significantly higher (*p* < 0.05) than the combination (6:2). However, the combination (7:1.5) showed an antagonistic effect (CI_avg_ > 1). The combination index (CI) for each combination usually has a different value at a different effect level. The plot of CI values at different effect levels could be obtained by CompuSyn software, which was named the Fa–CI plot. As an example, the Fa–CI plot for combination (6:1.5) was simulated by CompuSyn software and is shown in Figure 4D. The plot clearly illustrated that the CI value showed a decreasing trend with an increase in Fa value, indicating that the higher synergistic effect level was observed at the higher cellular antioxidant effect level for the EGCG and kaempferol combination. The results showed that the EGCG and kaempferol combination had synergistic antioxidant protection on the HepG2 cells in certain concentration ranges. According to the results, the dose–effect relationship of EGCG and kaempferol combinations was significant (*p* < 0.05). The CI value was lower than others, showing a significant synergistic effect. The combination of EGCG and kaempferol (6:1.5) was selected to conduct the follow-up experiment.

Previous studies reported that the EGCG and quercetin combination and the quercetin and kaempferol combination showed synergistic effects. Their synergistic mechanisms also illustrated that the combinations up-regulated the protein or genes of pathways to increase the capacity of scavenging reactive oxygen species (ROS) [20,24]. However, these results indicated that the level of synergism for each combination did not correlate with the antioxidant effect of individual compounds. The dose–effect relationship did not illustrate the mechanism. It only showed the mass-action law parameters, which is a complicated issue [26]. Based on the above results, the potential synergistic mechanism of EGCG and kaempferol combinations needed to be explored further.

### 2.4. Effect on Antioxidant Enzymes

The antioxidant enzyme system plays an important defensive role against oxidative stress injury. In order to evaluate whether the antioxidant enzyme system played a role in HepG2 cells, the activities of antioxidant enzymes (SOD, CAT, and GSH-Px), which play an important role in balancing oxidative stress in the human body [27], were measured. Therefore, changes in the activity of these intracellular antioxidant enzymes can reflect the capacity to inhibit the reactive oxygen species (ROS) in HepG2 cells.

To further illustrate the antioxidant mechanism of the EGCG and kaempferol combination (6:1.5), the activities of SOD, GSH-Px, and CAT were evaluated. The control was treated without compound and ABAP, while the negative control (ABAP group) was treated with ABAP but not compound. The results are shown in Table 2. The SOD, GSH-Px, and CAT activities of the ABAP group were 51.2 ± 3.1%, 53.5 ± 2.3%, and 57.1 ± 2.7% of the control group, respectively, which indicated that ABAP caused oxidant damage to HepG2 cells. When cells were pretreated with the EGCG and kaempferol combination (6:1.5) before ABAP treatment, however, the antioxidant enzymes activities increased compared to the ABAP group. The cells incubated with EGCG and kaempferol (1.5 + 0.375 μg/mL) showed an insignificant increase in SOD, CAT, and GSH-Px activities, while dramatically increased activity was found at higher concentrations. The CAT and GSH-Px activities were similar to those of SOD. The GSH-Px activities were increased by 7.6 ± 1.1%, 27.8 ± 1.3%, and 57.6 ± 2.4%, and the CAT activities were increased by 5.9 ± 0.2%, 24.2 ± 1.1%, and 42.1 ± 2.2% of the ABAP group value, respectively. The cells pretreated with EGCG (6 μg/mL) and kaempferol (1.5 μg/mL) showed a significant increase (*p* < 0.05) in SOD, CAT, and GSH-Px activities compared to the ABAP group. Similarly, the high concentration combination (3 + 0.75, 6 + 1.5 μg/mL) had better activities (*p* < 0.05) of SOD, CAT, and GSH-Px than did the individual EGCG or kaempferol. However, the low concentration of the combination (1.5 + 0.375 μg/mL) exhibited an insignificant increase (*p* > 0.05) in GSH-Px and CAT activities compared to EGCG or kaempferol. The results were consistent with the CAA assay. The compounds had better cellular antioxidant activities, and enzymes activities were higher.

**Figure 5:**
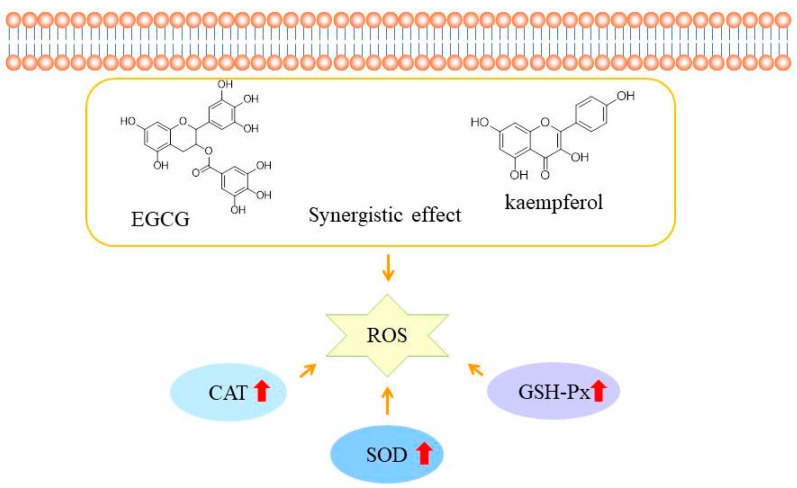
The possible mechanisms of EGCG and kaempferol combination antioxidant activities in HepG2 cells. The red arrow represent the activities of SOD, CAT, AND GSH-Px increase.

Based on the above results, the EGCG and kaempferol combination (6:1.5) could inhibit ABAP-induced oxidant stress in HepG2 cells by modulating the antioxidant enzymes activities. Moreover, the high concentrations of combinations showed the best activity of SOD, CAT, and GSH-Px in the sample groups. The results indicated that the appropriate concentration of combinations exhibited better antioxidant enzyme activity than the single compound, which suggested that the higher enzyme activity of combinations is the mechanism of synergy in the CAA assay. Wen et al. found that the cinnamtanin B1 from litchi leaf exhibited strong intracellular antioxidant activity by up-regulating the SOD, CAT, and GSH-Px activities [28]. Jiang et al. reported that the peptides from Jiupei had good cellular antioxidant activities, and the SOD, CAT, and GSH-Px activities increased in a dose-dependent manner [29]. Zhou et al. found that the antioxidant peptide from *Pinctada martensii* meat could significantly enhance the production of glutathione (GSH) and catalase (CAT) in HepG2 cells, as well as the expression of genes in the *Nrf2* signaling pathway [30]. Similarly, Huo et al. also reported that antioxidant peptides from Chinese baijiu exerted protective effects by scavenging the reactive oxygen species (ROS) and up-regulating cellular antioxidant enzyme (SOD, CAT, and GSH-Px) activities [31]. As shown in Figure 5, the EGCG and kaempferol combination can cross the cell membrane and increase the SOD, CAT, and GSH-Px activities to scavenge together the reactive oxidative species (ROS). Considering our results, we realize that the EGCG and kaempferol combination was more effective on the antioxidant enzymes in HepG2 cells. Therefore, the synergistic mechanism of the EGCG and kaempferol combination could be to up-regulate the higher SOD, CAT, and GSH-Px activities to achieve the effect of balancing the oxidative stress.

## 3. Materials and Methods

### 3.1. Chemicals and Reagents

Dimethyl sulfoxide (DMSO), 2′,7′-dichlorfluorescin diacetate (DCFH–DA), and 2,2-azobis (2-amidinopropane) dihydrochloride solution (ABAP) were purchased from Sigma-Aldrich (St. Louis, MO, USA). Epigallocatechin gallate (EGCG, HPLC grade, purity > 98%) and kaempferol (HPLC grade, purity > 98%) were obtained from Solarbio Science & Technology Co., Ltd. (Beijing, China). EGCG and kaempferol were dissolved in dimethyl sulfoxide (DMSO) at a concentration of 10 mg/mL and then freshly diluted in culture medium. The final concentration of DMSO in culture medium was below 0.05% (*v*/*v*). Phosphate buffer solution (PBS), minimum Eagle’s medium (MEM), fetal bovine serum (FBS), 0.05% trypsin–EDTA solution, penicillin (10,000 u/mL), and streptomycin (10,000 μg/mL) were purchased from HyClone (Logan, UT, USA). The Cell Counting Kit-8 (CCK-8) was obtained from Dojindo China Co., Ltd. (Shanghai, China). The BCA protein concentration test kit; cell lysis buffer for Western and IP; and phenylmethanesulfonyl fluoride (PMSF, 100 mM), superoxide dismutase (SOD), glutathione peroxidase (GSH-Px), and catalase (CAT) assay kits were purchased from Beyotime Biotechnology (Shanghai, China). All other chemicals and reagents with analytical grade used in this study were obtained from Sinopharm Group Chemical Reagents Co. Ltd. (Beijing, China).

### 3.2. Cell Culture

The HepG2 cell line was purchased from the Kunming Institute of Zoology, Chinese Academy of Sciences (Kunming, China). Briefly, HepG2 cells were cultured in minimum Eagle’s medium (MEM) at 37 °C with 5% CO_2_, along with 10% (*v*/*v*) FBS, and 1% (*v*/*v*) penicillin (1000 units/mL) and streptomycin (1000 μg/mL). Logarithmic growth phase cells were used for the follow-up experiments.

### 3.3. Cytotoxicity Assay

To determine the suitable concentrations of EGCG, kaempferol, and EGCG and kaempferol combinations for subsequent experiments, the cytotoxicity of EGCG, kaempferol, and their combinations was evaluated using the CCK-8 assay [32]. The HepG2 cells were cultivated with MEM medium mixed with 10% (*v*/*v*) FBS, and a 1% (*v*/*v*) penicillin–streptomycin solution in a humidified incubator containing 5% CO_2_ at 37 °C. The HepG2 cells were harvested by trypsinization and seeded into a 96-well microplate at a concentration of 4 × 10^4^ cells/well. The cells were pretreated with different concentrations of EGCG (0.625, 1.25, 2.5, 5, 10 μg/mL) and kaempferol (0.625, 1.25, 2.5, 5, 10 μg/mL) and with different combinations of EGCG and kaempferol (6:2, 6:1.5, 7:1.5, c/c) for 24 h. After being incubated for 24 h, the cellular morphologies were observed by microscope. The CCK-8 solution (10 μL) was added to the wells, and the absorbance of each well was measured at 450 nm by a microplate reader (SpectraMax i3x, Molecular Devices, LLC, San Jose, CA, USA). The cell viability was evaluated according to the above absorbance. The cell viability of the tested compounds was calculated as

Cell viability (%) = (1 − OD_sample_/OD_control_) × 100% (1)

where OD_sample_ is the absorbance of the well with compounds; OD_control_ is the absorbance of the control.

### 3.4. Cellular Antioxidant Activity (CAA) Assay

The CAA assay was conducted as described previously [33,34] with slight modification. HepG2 cells were seeded at 5 × 10^4^ cells/well in a 96-well microplate with 100 μL of growth medium/well. The outside wells of the plate were filled only with PBS in order to avoid the influence of the edge. After being incubated for 24 h, the growth medium was removed, and the wells were washed with PBS. Then, the cells were pretreated with different concentrations of EGCG (1, 2, 4, 8 μg/mL) and kaempferol (0.25, 0.5, 1, 2 μg/mL) and in different combinations (6:2, 6:1.5, 7:1.5, c/c) containing DCFH-DA (25 μM) for 1 h at 37 °C. After 1 h, the wells were washed thrice with PBS. Then 100 μL of 600 μM ABAP was added to the cells, and the 96-well microplate was immediately placed into the multifunctional microplate detector at 37 °C. The emission at 535 nm was detected with excitation at 485 nm every 5 min for 1 h. Control wells contained cells treated with DCFH-DA and ABAP. Blank wells contained cells treated with PBS without ABAP. The measured fluorescence values were converted into CAA units according to the following formula:

CAA unit (%) = (1 − ∫SA/∫CA) × 100

where ∫SA is the integrated area under the sample fluorescence versus time curve, and ∫CA is the integrated area from the control curve. The median effective dose (EC_50_) was measured for the median effect plot of log[(CAA unit/(1 − CAA unit)] versus log (dose).

### 3.5. Determination of Combination Index (CI) Values

To evaluate the interaction of EGCG and kaempferol combinations, the Chou–Talalay method, which was based on the median-effect equation, was used to measure the combination index (CI) values by describing the dose–effect curves of individual compounds and their combinations. Therefore, the EGCG and kaempferol interaction effects were evaluated as CI using CompuSyn software, CompuSyn, Inc., Paramus, NJ, USA [35]. In the present study, the combinations were composed of two compounds, and their CI values were evaluated by the CompuSyn software using the following formula: CI = (D)_1_/(D_X_)_1_ + (D)_2_/(D_X_)_2_, in which the (D_X_)_1_ and (D_X_)_2_ are the concentration, respectively, of sample 1 and sample 2 alone in which the cellular antioxidant effect is x%. Similarly, the (D)_1_ and (D)_2_ show the respective doses of sample 1 and sample 2, in which their combination antioxidant activity is x%. When CI = 1, the interaction between EGCG and kaempferol was an additive effect; when CI < 1, the interaction between EGCG and kaempferol indicated a synergistic effect, with the smaller CI showing the stronger synergistic effect; when CI > 1, the interaction between EGCG and kaempferol reflected an antagonistic effect [36].

### 3.6. Cellular Antioxidant Enzymes Activities of SOD, GSH-Px, and CAT

HepG2 cells were inoculated at a density of 1 × 10^6^ cells/well into the 6-well plate and cultured in a humidified incubator containing 5% CO_2_ at 37 °C for 24 h. After being washed thrice with PBS, the cells were treated with different concentration of EGCG (6 μg/mL), kaempferol (1.5 μg/mL), and a EGCG and kaempferol combination (6:1.5) for 1 h. After that, the cells were washed twice with PBS. Then the ABAP group was treated with 600 μM ABAP to induced oxidative stress, and the control group only contained an equal volume of MEM medium. All groups were then incubated for 1 h. The different groups were washed with PBS and then lysed with cell lysis buffer for Western blot and IP containing 1.0 mmol/L phenylmethane sulfonyl fluoride (PMSF) at 4 °C for 30 min. The lysed cells were centrifuged at 10,000× *g* for 10 min to gain the supernatant. Finally, the SOD, GSH-Px, and CAT assay kits were used to evaluated antioxidant enzyme activities. The protein concentrations were quantified using a BCA protein concentration test kit [37].

### 3.7. Statistical Analyses

All the experiments were performed in triplicate (n = 3), and data were reported as mean ± standard deviation (SD). The Duncan test of one-way ANOVA was used to analyze the significant differences among results. The significant difference was set as *p* < 0.05.

## 4. Conclusions

In this study, we found that EGCG and kaempferol combinations (6:1.5, 6:2) had synergistic cellular antioxidant activity in HepG2 cells. Moreover, these combinations protected HepG2 cells against ABAP-induced oxidative stress by scavenging ROS and up-regulating higher cellular antioxidant enzyme activities, including SOD, CAT, and GSH-Px, in a dose-dependent manner. Accordingly, this research presents the feasibility of combined dietary flavonoids, which show some beneficial functions with synergistic effects. The synergistic antioxidant effects of EGCG and kaempferol combinations provide a theoretical basis for the development of functional food ingredients.

## Figures and Tables

**Table 1:** The CI values of EGCG + kaempferol on cellular antioxidant activity.

Compound	Proportion (c/c)	CI			CI_avg_
		GI_50_	GI_75_	GI_90_	
EGCG + kaempferol	6:2	1.07 ± 0.02	0.89 ± 0.01	0.74 ± 0.01	0.85 ^a^
	6:1.5	0.58 ± 0.01	0.55 ± 0.02	0.52 ± 0.01	0.54 ^b^
	7:1.5	>1	>1	>1	>1

CI values of each compound are presented at 50%, 75%, and 90% of antioxidant effect (GI_50_, GI_75_, and GI_90_). CI_avg_ = (CI_50_ + 2CI_75_ + 3CI_90_)/6, is the weighted average CI, and CI < 1, CI = 1, and CI > 1 represent synergism, additive effect, and antagonism, respectively. The results are represented as means ± SD (n = 3). The values having no letters in common are significantly different (*p* < 0.05).

**Table 2:** Effect of EGCG + kaempferol (6:1.5) on activities of antioxidant enzymes in the presence of ABAP.

Compound (μg/mL)	SOD (U/mg Protein)	GSH-Px (mU/mg Protein)	CAT (U/mg Protein)
control	29.3 ± 2.2 ^a^	310.5 ± 5.4 ^a^	61.3 ± 3.1 ^a^
ABAP	14.3 ± 1.1 ^f^	133.3 ± 2.3 ^e^	28.5 ± 2.2 ^e^
1.5 + 0.375	15.2 ± 0.8 ^e^	143.4 ± 4.1 ^d^	30.2 ± 1.2 ^d^
3 + 0.75	18.2 ± 1.3 ^c^	170.3 ± 3.7 ^c^	35.4 ± 2.4 ^c^
6 + 1.5	22.6 ± 1.5 ^b^	210.1 ± 4.8 ^b^	40.5 ± 2.5 ^b^
6	16.5 ± 1.2 ^d^	150.2 ± 2.5 ^d^	31.3 ± 1.3 ^d^
1.5	15.4 ± 0.6 ^e^	148.4 ± 2.5 ^d^	30.5 ± 0.5 ^d^

Different letters represent significant differences at *p* < 0.05. The initial concentrations of EGCG and kaempferol were 6 and 1.5 μg/mL, respectively.

## Data Availability

The data presented in this study are available upon request from the corresponding author.
